# Divergent HIV and Simian Immunodeficiency Virus Surveillance, Zaire

**DOI:** 10.3201/eid1109.050179

**Published:** 2005-09

**Authors:** Amanda Schaefer, Kenneth E. Robbins, Eugene Nzila Nzilambi, Michael E. St. Louis, Thomas C. Quinn, Thomas M. Folks, Marcia L. Kalish, Danuta Pieniazek

**Affiliations:** * Centers for Disease Control and Prevention, Atlanta, Georgia, USA;; † Project SIDA, Kinshasa, Democratic Republic of Congo;; ‡ National Institutes of Health, Bethesda, Maryland, USA

**Keywords:** HIV-1, HIV-2, SIV, Zaire, surveillance, dispatch

## Abstract

Recent HIV infection or divergent HIV or simian immunodeficiency virus (SIV) strains may be responsible for Western blot–indeterminate results on 70 serum samples from Zairian hospital employees that were reactive in an enzyme immunoassay. Using universal polymerase chain reaction HIV-1, HIV-2, and SIV primers, we detected 1 (1.4%) HIV-1 sequence. Except for 1 sample, no molecular evidence for unusual HIV- or SIV-like strains in this sampling was found.

HIV-1 and HIV-2 are believed to be the result of cross-species transmission from simian immunodeficiency virus (SIV)–infected chimpanzees and sooty mangabeys, respectively, which represent 2 (SIVcpz and SIVsm) of the 6 major lentiviral phylogenetic lineages (*1**,**2*). No evidence exists that SIV strains from the remaining nonhuman primate lineages have infected humans, although many grow in human cells in vitro as do SIVcpz and SIVsm (*3*). Since humans are exposed to a plethora of primate lentiviruses through blood or body fluids during hunting, butchering, eating bushmeat, and keeping primates as pets (*3*), the potential exists for zoonotic transmission of diverse primate lentiviruses in many parts of sub-Saharan Africa, including the Congo River basin (*4*). This potential is supported by the identification of a Cameroonian man whose HIV serologic results were indeterminate but whose serum specimen reacted strongly and exclusively with an SIVmnd V3 loop peptide (*5*). An even more compelling case for cross-species exposure is the recent finding of a Cameroonian man who may have been exposed to a colobus SIV, as indicated by a strong humoral (*env* IDR and V3) and a weak cellular (*gag*) immune response (*6*). Although SIV sequences were not identified in either case, the findings suggest that humans are probably exposed to different simian retroviruses that can establish new infections in humans (*3*).

Nonhuman primates infected with SIV from the currently recognized lineages can harbor antibodies that serologically cross-react with some HIV-1 or HIV-2 antigens (*3*). In many cases, HIV Western blots (WBs) with indeterminate profiles of SIV-infected monkeys resemble those of HIV enzyme immunoassay (EIA)–positive, WB-indeterminate human sera from Africa. In general, such indeterminate African sera demonstrate a broad range of reactivity to HIV-1 proteins, in contrast to predominant p24 reactivity in WB-indeterminate sera from persons in the United States (*7**–**9*). These data suggest that the WB-indeterminate patterns in HIV EIA-reactive sera from persons living in Africa may reflect more than just a recent HIV infection or an infection with a highly divergent HIV-1 strain (*10*); they may reflect either cross-reactivity with unknown pathogens of African origin or exposure to new HIV- or SIV-like strains.

## The Study

We investigated the presence of HIV or SIV in 70 HIV EIA-reactive, WB-indeterminate serum specimens collected in 1984 and 1986 from employees of Mama Yemo Hospital in Kinshasa, Zaire (currently the Democratic Republic of Congo) (*11*). WB bands were identified by using an HIV-1/2 WB assay (version 2.2, Genelabs Diagnostics, Singapore) and classified as indeterminate according to criteria for interpreting HIV-1 (http://www.cdc.gov/mmwr/preview/mmwrhtml/00001431.htm). Briefly, a positive WB result indicates reactivity to at least p24 and 1 of the 3 *env* (gp41, gp120, or gp160) proteins with banding intensity at least as strong as that seen in the weak-positive control; a negative WB result indicates no reactivity and an indeterminate WB result indicates reactivity to at least 1 protein.

Of the 70 WB-indeterminate serum specimens, 69 showed a broad range of HIV-1 WB-indeterminate band patterns, and 1 had an HIV-2 WB-indeterminate pattern with weak reactivity against gp36, p68, and gp80 proteins. Analysis of HIV-1 WB-indeterminate patterns provided several important observations. First, most of the specimens (41/69, 59%) showed reactivity to multiple viral proteins (Figure), whereas the remaining specimens (28/69, 41%) demonstrated reactivity against single HIV-1 proteins, including p24 (13/69, 18.8%), p17 (n = 8, 11.6%), gp160w (w = weak) (n = 4, 5.8%), and p51, p66, and gp41w (n = 1 each; 1.4% each). Second, in all but 3 specimens the WB-indeterminate patterns with reactivity to multiple viral proteins combined proteins of p24 or p17 gag or both with others, giving the following 21 profiles: p17/p24 (14/69, 20.3%); p24/p66 and p17/p24/p66 (n = 3 each, 4.3% each); p24/p51, p17/p24/p51, and p17/p24/gp41w (n = 2 each, 2.9% each); p17/p66, p17/gp160, p24/gp160, p24/p51/gp41w, p17/p51/p66, p17/p31/gp41w, p24/p51/p55, p24/p51/p66, p17/p24/p51/p66, p17/p24/p51/p55, p17/p24/p55/p66, p24/p31/p51/p55, p31/p66, p51/gp41w, and p31/p66/gp41w (n = 1 each, 1.4% each). Overall, this analysis indicated reactivity to 8 HIV-1 proteins that occurred as single bands or multiple combinations. Whereas reactivity to p24 might represent either early infection with HIV or reaction to nonspecific antigens (*8*), reactivity to other HIV-1 proteins (p17, p31, p51, p55, p66, gp41, or gp160) could reflect at least exposure to HIV-1, HIV-2, or SIV variants as well as cross-reactivity with yet unidentified HIV- or SIV-like strains (*3**,**10*).

**Figure:**
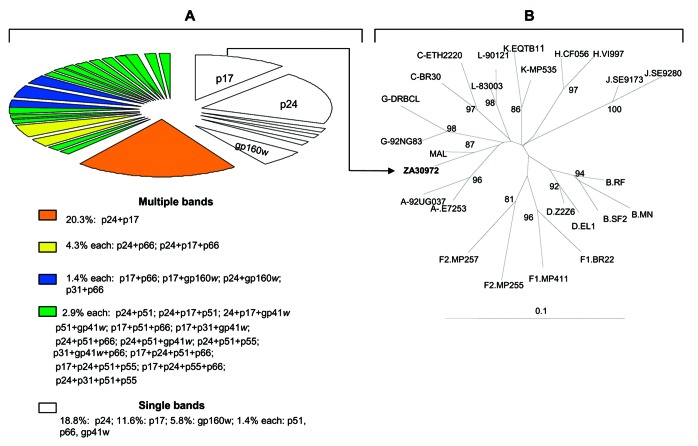
A) Distribution of HIV-1 Western blot–indeterminate patterns among 69 serum specimens from Kinshasa, Zaire, reactive by enzyme immunoassay. B) Phylogenetic classification of HIV-1 protease sequence ZA30972 (GenBank accession no. AY562558) isolated from the p17 gag-reactive serum. The phylogenetic tree was generated by the neighbor-joining method with the nucleotide distance calculated by Kimura 2-parameter method (*12*), included in the GeneStudio package (http://www.genestudio.com). Reference sequences were obtained from the Los Alamos database (http://hiv-web.lanl.gov/MAP/hivmap.html). The position of the outgroup (Simian immunodeficiency virus [SIV]cpz) is not shown. Values on branches represent the percentage of 100 bootstrap replicates. The scale bar indicates an evolutionary distance of 0.10 nucleotides per position in the sequence.

To determine whether HIV- and SIV-like RNA could be detected in the 70 WB-indeterminate specimens, we attempted reverse transcription–polymerase chain reaction (RT-PCR) amplification of HIV-1, HIV-2, and SIV. However, because of a small volume (<300 mL) of some serum specimens, a limited number of primers was used. We selected 4 sets of primers for 3 highly conserved gene regions, including *env*-gp41, *pol* protease, and *pol* integrase, which we previously developed and extensively tested for the efficient PCR amplification of field specimens. Briefly, gp41 primers allow the amplification of HIV-1 groups M (subtypes A–K and U [unclassifiable]) N, and O and SIVcpz with an efficiency of 95% (*13*); HIV-1 type specific protease primers efficiently (>95%) detect group M viruses (subtypes A–K and U [unclassifiable]), and allow successful amplification of some PCR–gp41 negative specimens. HIV-2 type-specific protease primers allow the amplification of HIV-2 at least subtypes A and B (*13*), and HIV-2/SIV integrase primers amplify HIV-2 and at least 5 major SIV lineages including SIVcpz, SIVsm, SIVagm (African green monkey), SIVmndBK12 (mandrill), and SIVsyk (Sykes monkey) (*14*). The integrase primers should also amplify other SIV lineages including SIVcol, SIVLhoest/SIVsun, SIVgsn, SIVmus, SIVmon, SIVtal, and SIVdeb (*3*). This prediction is based on the fact that genetic diversity of these sequences within the 3´-end of primer regions was the same as in strains previously used for testing (*15*), which was clearly visible through DNA alignment of the integrase primers with all SIV genomic integrase sequences deposited in GenBank. Positive controls for PCR amplification included HIV-1 Mn, HIV-2 ROD, and SIVsm. Using this approach, we amplified a 297-bp HIV-1 protease (PR) gene from only 1 (1.4%) of the 70 serum specimens. The remaining 69 specimens were PCR negative for all the primers tested. Phylogenetic analysis of the PR gene sequence showed a close phylogenetic relationship with the HIV-1 MAL strain (Figure), a recombinant virus that contains portions of subtypes A and D and an unclassifiable region that was identified in 1985 in Zaire (*16*).

## Conclusions

The PR gene sequence was identified in the serum of the person with reactivity to only the p17 *gag* band by WB, which suggests that this person was recently infected and that antibodies to all the HIV-1 antigens had not yet developed. No information was available on demography, risk, or clinical status of this person. In the remaining 69 WB-indeterminate specimens, we could not rule out exposure to SIV from nonhuman primates, from handling infected animal meat before consuming it, or from keeping monkeys as pets. Although the limited molecular scope of this study and the quality of the old serum specimens may not be adequate for molecular confirmation of such viruses, our findings of complex HIV WB-indeterminate patterns with reactivity to multiple viral proteins in serum specimens from persons living in the Democratic Republic of Congo provide comprehensive insights into HIV WB-indeterminate sera in the mid-1980s.
